# A Simple, Anatomical, and Reproducible Technique for Harvesting a Partial-Thickness Layered Rectangular Quadriceps Tendon–Bone Autograft: Using Standard Surgical Instruments

**DOI:** 10.1016/j.eats.2025.103918

**Published:** 2025-10-10

**Authors:** Yasukazu Yonetani, Kazunori Shimomura, Akira Tsujii, Ayaka Tanaka, Ryo Miyazaki, Hidekazu Suzuki, Masayuki Hamada

**Affiliations:** a Osaka Nakanoshima Orthopedic Clinic, Osaka, Japan; b Department of Sports Orthopaedics, Japan Community Health Care Organization Hoshigaoka Medical Center, Osaka, Japan; c Department of Rehabilitation, Kansai University of Welfare Sciences, Osaka, Japan; d Department of Orthopaedic Surgery, Japan Community Health Care Organization Hoshigaoka Medical Center, Osaka, Japan; e Department of Sports Medical Biomechanics, The University of Osaka Graduate School of Medicine, Osaka, Japan; f Department of Orthopaedic Surgery, The University of Osaka Graduate School of Medicine, Osaka, Japan; g Department of Orthopaedic Surgery, Seifu Hospital, Osaka, Japan

## Abstract

Anatomic anterior cruciate ligament reconstruction (ACLR) aims to replicate the native 3-dimensional architecture of the anterior cruciate ligament (ACL), including its fiber orientation and insertion site, to restore normal knee biomechanics. The anatomic rectangular tunnel ACLR technique, pioneered by Shino et al., creates rectangular tunnels that better replicate the native ACL's fiber alignment compared with conventional round tunnels. Recently, the quadriceps tendon−bone autograft has gained attention because of its large cross-sectional area, strong tensile properties, and lower incidence of anterior knee pain compared with bone−patellar tendon−bone grafts. This technical note describes a layered quadriceps tendon−bone harvesting technique that preserves the third tendon layer while harvesting a sufficient graft with a rectangular bone block. The method uses minimal instruments and relies on anatomical landmarks to ensure reproducibility and graft quality. Benefits of this technique include anatomical reconstruction of the ACL, decreased graft-tunnel mismatch, potential for reduced tunnel widening, and favorable healing as the result of bone-to-bone integration. Limitations include the technical complexity of tendon harvesting, risk of patellar fracture, and a steep learning curve. With appropriate anatomical knowledge and surgical experience, this approach can be a reliable and effective option for anatomic ACLR.

Anterior cruciate ligament (ACL) reconstruction seeks to restore joint stability by replicating the anatomical and biomechanical properties of the native ligament. Shino et al.’s^1^, ^2^, ^3^, ^4^, ^5^, ^6^ anatomic rectangular tunnel anterior cruciate ligament reconstruction (ART-ACLR) allows graft placement in line with the natural ACL fiber direction, improving functional outcomes and reducing graft stress. Mimicking the native bone−tendon junction and fiber arrangement is essential for long-term success.

Quadriceps tendon−bone (QTB) grafts have demonstrated comparable strength and superior cross-sectional area compared with bone−patellar tendon−bone (BPTB) grafts,^6^, ^7^, ^8^, ^9^, ^10^ with less associated anterior knee pain.^6^^,^^11^, ^12^, ^13^, ^14^ Their application within the ART technique may further optimize biomechanical and clinical outcomes.^6^

## Surgical Technique

### Surgical Indications and Contraindications

This technique is applicable to all patients requiring ACL reconstruction. Contraindications include a history of quadriceps tendon (QT) rupture or tendinopathy and bipartite patella, which can complicate bone-block harvesting.

### Positioning

The surgery is performed after induction of general or regional anesthesia. The patient is placed supine with the knee flexed in either the leg-hanging or lithotomy position with a nonsterile high thigh tourniquet. The QT is best harvested under tension.

### Technique to Harvest Layered QT Graft With Rectangular Patellar Bone Block

**Fig 1:**
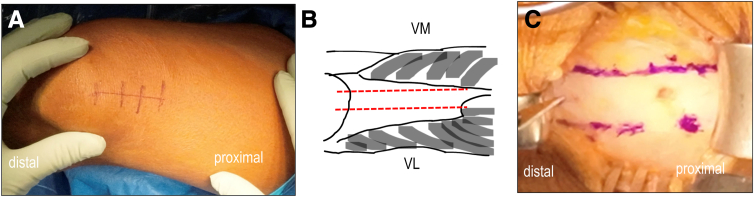
Quadriceps tendon−bone harvest—Step 1. (A) A 4- to 5-cm skin incision from patella proximal pole to proximal thigh. (B) Schematic showing the anatomy of quadriceps tendon, muscle bellies (vastus medialis: VM, vastus lateralis: VL), and harvest site (red dashed line). (C) marking the 2 harvest margins 10-mm apart (A-C): Right knee, supine position.

A 4- to 5-cm vertical skin incision is made proximally from the upper edge of the patella (Fig 1). Skin mobility is assessed before incision to ensure proper exposure.

Blunt dissection is used to expose the QT. The optimal graft site is approximately 2 mm medial to the patellar midline^15^ (Fig 1B). An understanding of this anatomy is essential before operating, as tendon thickness and location vary.^15^ The central tendon is approximately 10 mm thick and 80 mm long,^16^ but in our experience, graft lengths average 60 mm in women and 70 mm in men.

After exposing the QT and underlying muscles (vastus medialis/vastus lateralis), the synovial bursa is opened longitudinally to visualize the entire harvesting area. Using a 10-mm osteotome, the harvest site is marked longitudinally (Fig 1B). The distal marking is centered 2 mm medial from the patellar midline, and the proximal limit is defined just adjacent to the vastus medialis muscle belly (Fig 1C).

**Fig 2:**
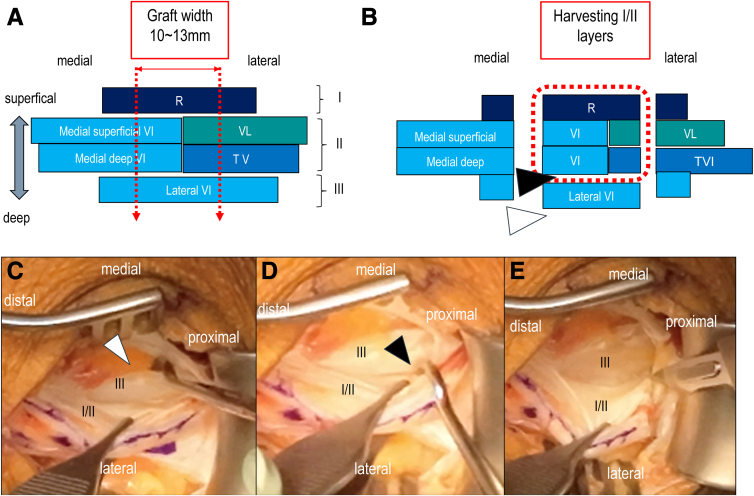
Quadriceps tendon−bone harvest—Step 2. (A) Schematic showing 3-layered QT structure, with cutting line (red dashed arrow). (B) Schematic showing harvest of the QT tendon (red dashed square) and position of hook for layer separation to identify layer III and II. (C) Superficial I/II layer grasped with forceps, and layer III identified with hook (white arrowhead). (D) the space between layer I/II and layer III identified and separated with hook (black arrowhead). (E) Harvest tendon dissected from deep side to avoid the damage of layer III. (QT, quadriceps tendon; R, rectus femoris; VL, vastus lateralis; VI, vastus intermedius; TVI, tensor vastus intermedius.)

The QT consists of 3 layers: layer I (rectus femoris), layer II (medial/lateral vastus intermedius: VI), and layer III (deep VI)^17^ (Fig 2A). Our technique isolates and preserves layer III, harvesting only layers I and II (Fig 2B). An No. 11 blade scalpel is used to incise the tendon longitudinally along the markings, starting medially. Incision depth should reach the joint capsule to prepare for layered dissection. The graft length is confirmed with a K-wire or ruler. Then, the interface between layers II and III is identified using blunt hooks (Fig 2C-E). This “cleavage plane” allows safe separation of the superficial layers^15^ (Fig 2B). Sharp dissection is used only when necessary to avoid damaging the graft.

**Fig 3:**
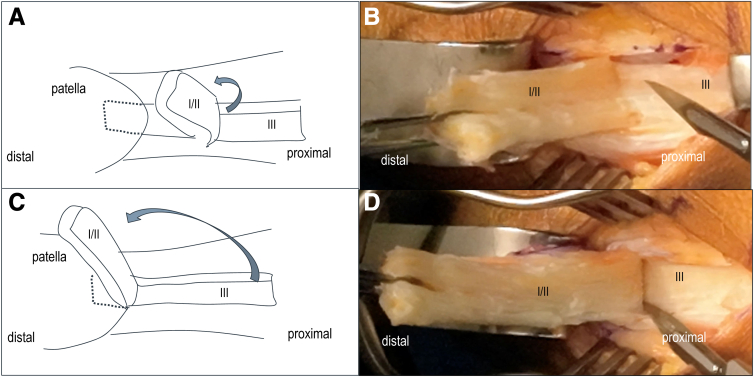
Quadriceps tendon−bone harvest—Step 3. (A-B) Manually peel off layers I/II from layer III, sometime assisting with a scalpel. (C-D) Expose the patellar cortex at bone-tendon junction level.

Once dissection is complete, the tendon is peeled distally toward the patella by grasping the graft with forceps and slowly detaching it, similar to peeling a string cheese (Fig 3). This is done manually whenever possible. It is crucial that both medial and lateral incisions fully reach the capsule; otherwise, dissection becomes difficult.

**Fig 4:**
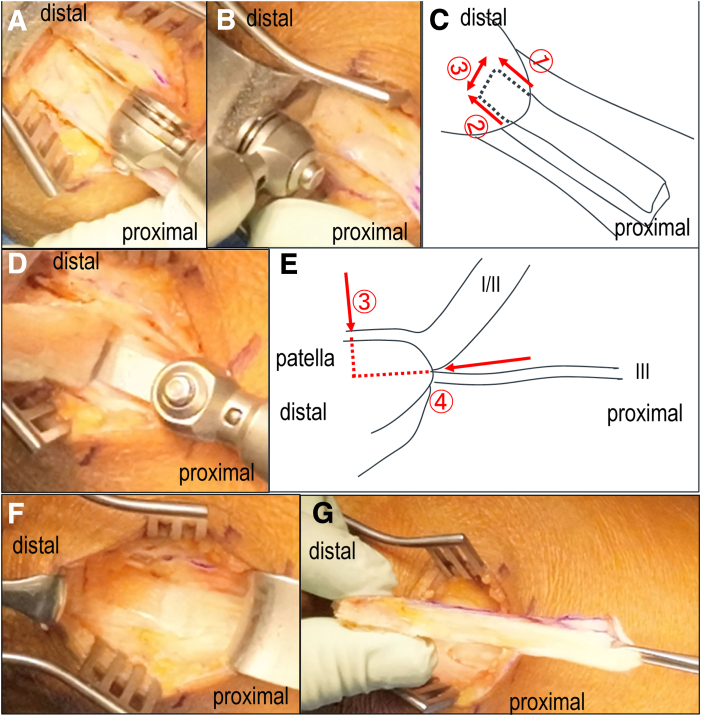
Quadriceps tendon−bone harvest—Step 4. (A) Vertical osteotomy from proximal to distal at marking a 10- × 15-mm bone plug avoiding excessive bone cut. (B) distal osteotomy avoiding joint penetration with stopper of oscillating saw. (C) Preserving layer III and executing shallow cuts parallel to patella surface. (D) Coronal osteotomy from proximal to distal paralel to the patellar surface. (E) Schematic lateral sequence of coronal osteotomy at proximal of patella. (F) small cubic patella bone defect and preserved layer III. (G) harvested rectangular quadriceps tendon−bone graft.

When the distal end reaches the patellar border, the graft is gently tensioned, and a 10- × 15-mm bone plug is outlined with cautery (Fig 4A–E). Three-way bone cuttings are performed using a 10-mm oscillating saw (Stryker), sagittal cutting is from proximal to distal staying vertical to patella surface (Fig 4A), coronal-cutting is from proximal to distal staying parallel to the patellar surface (Fig 4D). Using a bone saw and osteotome, the plug is carefully removed while minimizing bone depth to avoid fracture (Fig 4A-E).^18^

After completing side, distal, and coronal cuts, the proximal bone is gently elevated using a chisel. Care is taken to extract only the bone attached to the harvested tendon, minimizing donor-site damage (Fig 4F). The graft is then removed (Fig 4G). The joint capsule (layer III) is not routinely closed. Subcutaneous tissue is reapproximated loosely to allow for drainage. The surgical field is then prepared for intra-articular procedures.

### Graft Preparation

**Fig 5:**
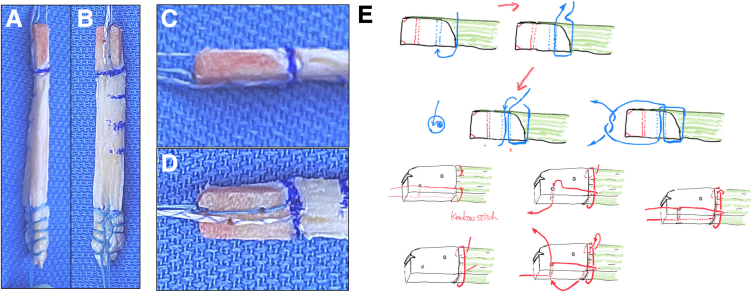
Graft preparation: processed QTB graft (A); enlarged bone plug view (B); suture technique for AM (C) and PL (D) ends to make it easy to insert into the bone tunnel. (E) Shenmatic sequ Strong suture passed through the bone−tendon junction. Each suture end passed through bone tunnel made by 1.5-mm K-wire. Finally suture tied up the end of bone plug for easy implantation. (D) One Krakow stich placed at bone−tendon junction for backup of bone plug breakage. Each suture end passed through the bone tunnel as same manner as AM ends. (AM, anteromedial; PL, posterolateral; QTB, quadriceps tendon−bone.)

The bone plug is drilled in 2 places, 5 and 10 mm from the bone−tendon junction (Fig 5B). Two strong sutures (No. 2 FiberWire; Arthrex) (XBraid TT 1.2 mm; Stryker) are passed through each drill hole and bone tendon junction (Fig 5 C and D). The passed suture is tied at the small cut of bone plug tip for pull-through (Fig 5C). The soft tissue end of the graft is sutured with strong sutures (XBraid TT 2.0 mm; Stryker), using a Krakow suture technique (Fig 5A). Tips and pearls for performing this technique are summarized in the Table 1.

**Table 1:** Pearls and Pitfalls

Pearls	Pitfalls
Identify the proper tissue, not only superficial layer, but also muscle belly both of vastus medialis and vastus lateralis.	Lateral part of the quadriceps tendon leads not only thinner tendon, but also thin part of patella bone.
During guiding where to cut the quadriceps tendon, the medial border of the osteotome has to be localized as close as possible not only to the vastus medialis muscle belly at proximal end, but also to the 7.5-mm medial from central of patella pole at distal end because of its thickest part of quadriceps tendon at this way.	Because of discontinuity of tendon fiber after another blunt harvesting technique, substantially weaken may occur at the whole graft strength.
When cutting the quadriceps tendon and patella, cuts need to be perpendicular to the patellar and quadriceps tendon surface. This tips achieve rectangular parallelepiped shape on all of the graft's length.	
Longitudinal cutting need for complete opening joint capsule. This tips make it easy to confirm the space between superficial I/II layers and third layer.	
Harvest layered tendon keep continuous fibers from proximal end to distal end.	
To avoid patellar fracture, use half part of a 10-mm oscillating saw. After marking the place of planned patella osteotomy with the electric knife, the patellar osteotomy performed directing from the proximal part of the patella and parallel to the anterior surface of the patella.	
No suturing to tendon itself avoids excessive tension to patella-femoral joint.	

## Discussion

This technique addresses 2 major challenges in anatomical rectangular ACL reconstruction: safe and reproducible QTB harvesting and precise minimum size harvesting for using rectangular tunnel methodology. Our approach requires no proprietary harvesting devices, improves graft size consistency, minimizes patellar morbidity, and enhances anatomical replication. Previous studies demonstrate comparable outcomes between BPTB and QTB grafts, with reduced anterior knee pain in QTB patients.^6^

In contrast to full-thickness harvesting techniques—which remove the entire tendon thickness including layer III—our sublayered method preserves deeper structures to maintain extensor mechanism integrity and avoid weakening the bone–tendon junction. Malinowski et al.^19^ presented a full-thickness QTB harvest using basic tools, reporting reliable graft quality and suitability for both ACL and posterior cruciate ligament reconstruction. Although the full-thickness approach provides robust graft size and strength, it requires meticulous closure of the donor site to prevent intra-articular hematoma and may increase the risk of postoperative quadriceps atony or patellar fracture if performed without appropriate depth control.^18^

Our layered approach offers a balance between safety and graft strength. By harvesting only layers I and II and preserving layer III, the procedure minimizes surgical morbidity and allows for repeatable graft quality without compromising patellar bone stability. In addition, our method facilitates partial-thickness harvesting in patients with smaller tendon dimensions, thereby expanding its applicability.

Despite being technically demanding in early learning phases,^20^ the layered technique allows surgeons to maintain anatomical structure, reduce postoperative pain, and still achieve adequate graft dimensions. Moreover, the graft obtained is well-suited for insertion into rectangular tunnels that mimic native ACL fiber orientation, promoting better biomechanical function and bone−tunnel healing.^1^^,^^4^^,^^5^^,^^21^^,^^22^

Future research should directly compare layered versus full-thickness QTB harvesting techniques in terms of biomechanical strength, clinical outcomes, and donor-site complications. The choice of method should be tailored to patient anatomy, surgical expertise, and procedural goals.

Most layered QTB graft disadvantages are associated with its harvesting: both sophisticated instruments and procedures without reference to anatomical characteristics of quadriceps tendon.^14^^,^^23^, ^24^, ^25^ Our simple technique makes it easier to harvest layered QTB on the basis of the anatomical features, the strongest and multipotential graft. Advantages and disadvantages of the technique are summarized in the Table 2.

**Table 2:** Advantages and Disadvantages of Rectangular Layered Quadriceps Tendon−Bone Autograft Harvesting Technique

Advantages	Disadvantages
The strongest graft option.	Joint cavity has to be opened.
20% more collagen fibers than bone−patellar tendon−bone with the same cross-section.	Irrigation solution increase rather than another graft because of opening joint cavity.
1.5∼2.0 times of cross-sectional area than bone−patellar tendon−bone graft	Possibility of occurrence and subcutaneous penetration of intra-articular hematomas exist at donor place.
Use of bone block grafts eliminates the risk of weakening the graft on the edge of the femoral bone tunnel.	Postoperative transient atony of the quadriceps muscle is observed.
Additional graft in multiligament reconstructions.	Risk of patellar fracture.
Adjustability of graft length and no risk of mismatch.	Tourniquet has to be used.
Lower risk of anterior knee pain, patellar ligament shortening, and sensory loss than in bone−patellar tendon−bone.	
Muscular agonists of anterior cruciate ligament are not harvested as in hamstring tendons graft.	
Minimum size of bone block, very useful in revision cases to avoid overlapping previous tunnel position	
Bone block and tendon with a rectangular cross-section perfectly fitted to the prepared femoral and tibial rectangular tunnel, minimize the graft−tunnel mismatch.	
Direct healing of the bone block with bone tunnel.	
Osteointegration is faster than in hamstring tendons graft.	
Quadriceps tendon−bone is suitable for posterior cruciate ligament reconstruction because longer graft is necessary,	
Only standard surgical instruments are needed.	
The bone−tendon junction is not weakened.	

## Disclosures

All authors (Y.Y., K.S., Ak.T., Ay.T., R.M., H.S., M.H.) declare that they have no known competing financial interests or personal relationships that could have appeared to influence the work reported in this paper.

## Supplementary Data

Video 1 This video demonstrates a simple, anatomical, and reproducible technique for harvesting a layered rectangular quadriceps tendon–bone autograft using standard surgical instruments. A 4- to 5-cm vertical line is marked, starting from the center of the patella. We check the mobility of the skin to make sure the upper half of the patella can be fully exposed. After making the skin incision, the subcutaneous tissue and paratenon are carefully cut to reveal the white surface of the quadriceps tendon and the muscle underneath. A 10-mm-wide osteotome is placed perpendicular to the patella, midway between the vastus medialis and vastus lateralis muscle bellies. Two straight lines, 10 mm apart, are drawn along the osteotome. The medial line should be placed as close as possible to the vastus medialis. The tendon is then cut along the fibers, extending about 5 to 6 centimeters above the patella. A retractor is inserted at the cut edge to expose all three layers of the tendon. Using a blunt hook, we can easily identify the space between the second and third layers, and begin gentle separation. Once the first and second layers are grasped with forceps, they are carefully dissected from the joint capsule using a scalpel. After completing the transverse cut, the graft is tensioned using the surgeon's finger and tendon forceps, and then gently released from the distal end. Before fully detaching the tendon, we recommend measuring the cross-sectional area. If it's too small, the plan can be changed to a full-thickness harvest. The tendon is then peeled—by hand or with a knife—in a way that resembles pulling string cheese. Medial and lateral incisions are made down to the cortex of the upper pole of the patella to define the cutting lines. A 10- by 15-mm bone block is marked on the anterior surface of the patella. With the tendon under tension, an oscillating saw with a 10-millimeter blade is used to make sagittal cuts on both sides, and a transverse cut 15 mm distal to the top of the patella. Finally, an oscillating saw is placed the marked line, and with the tendon pulled upward and the oscillating saw aligned parallel to the patellar surface. Two or three gentle taps with osteotome are used to complete the osteotomy. The final result is a layered quadriceps tendon–bone autograft, harvested through a 5-centimeter skin incision. As you can see, the patellar bone defect is minimal, and the third layer of the tendon is preserved. This technique offers a reliable and efficient way to harvest a rectangular quadriceps tendon with a bone block—using only simple tools
